# Effects of Inter-Stimulus Interval on Speech-Evoked Frequency-Following Response in Elderly Adults

**DOI:** 10.3389/fnagi.2018.00357

**Published:** 2018-11-08

**Authors:** Dongxin Liu, Jiong Hu, Ruijuan Dong, Jing Chen, Gabriella Musacchia, Shuo Wang

**Affiliations:** ^1^Otolaryngology—Head & Neck Surgery, Beijing Tongren Hospital, Beijing Institute of Otolaryngology, Capital Medical University, Beijing, China; ^2^Department of Audiology, University of the Pacific, San Francisco, CA, United States

**Keywords:** evoked potential, frequency-following response, lexical tone, aging, inter-stimulus intervals

## Abstract

**Background**: The speech-evoked frequency following response (FFR) has shown to be useful in assessing complex auditory processing abilities and in different age groups. While many aspects of FFR have been studied extensively, the effect of timing, as measured by inter-stimulus-interval (ISI), especially in the older adult population, has yet to be thoroughly investigated.

**Objective**: The purpose of this study was to examine the effects of different ISIs on speech evoked FFR in older and younger adults who speak a tonal language, and to investigate whether the older adults’ FFR were more susceptible to the change in ISI.

**Materials and Methods**: Twenty-two normal hearing participants were recruited in our study, including 11 young adult participants and 11 elderly participants. An Intelligent Hearing Systems Smart EP evoke potential system was used to record the FFR in four ISI conditions (40, 80, 120 and 160 ms). A recorded natural speech token with a falling tone /yi/ was used as the stimulus. Two indices, stimulus-to-response correlation coefficient and pitch strength, were used to quantify the FFR responses. Two-way analysis of variance (ANOVA) was used to analyze the differences in different age groups and different ISI conditions.

**Results**: There was no significant difference in stimulus-to-response correlation coefficient and pitch strength among the different ISI conditions, in either age groups. Older adults appeared to have weaker FFR for all ISI conditions when compared to their younger adult counterparts.

**Conclusion**: Shorter ISIs did not result in worse FFRs from older adults or younger adults. For speech-evoked FFR using a recorded natural speech token that is 250 ms in length, an ISI of as short as 40 ms appeared to be sufficient and effective to record FFR for elderly adults.

## Introduction

The frequency-following response (FFR) recorded from the human scalp is an electrophysiological potential which follows the periodicity of the stimuli (Moushegian et al., 1973). It is a far-field potential recorded from surface electrodes, reflecting the synchronous activity of axonal and dendritic potentials generated primarily by populations of neurons in the lateral lemniscus and inferior colliculus of the brainstem (Smith et al., 1975; Møeller, 1998). In recent years, the FFR has seen a renewed interested in the research realm due to its unique ability to assess various auditory functions using complex stimuli (Skoe and Kraus, 2010). One reason that the FFR has been successful in this regard is that the frequency information that is extracted from the FFR waveform corresponds to the spectrum of the stimulus. By examining the spectral property of FFR, one can have a means to assess the processing of complex sounds in the auditory system (Cunningham et al., 2001; Aiken and Picton, 2008; Skoe and Kraus, 2010; White-Schwoch et al., 2013). For example, Krishnan et al. (2004, 2005) used four different Mandarin Chinese tones to evoke FFR in young adult native speakers of Mandarin to examine the effect of language background on pitch processing. Another application of FFR is to use a consonant-vowel (CV) complex, typically /da/, to evaluate various aspects of the auditory processing function, such as the effect of maturation (Anderson et al., 2010), auditory training (Song et al., 2008), music training (Musacchia et al., 2008), language and/or reading issues (Banai et al., 2009) and speech perception in noise (Banai et al., 2009). Germane to the current study, the FFR has been utilized to assess how aging affects the physiological process of auditory function. For example, Clinard et al. (2010) demonstrated that older adults, even with normal hearing sensitivity, have auditory perceptual deficits relative to their young counterparts. Similarly, Wang et al. (2016) demonstrated that pitch processing ability at the brainstem level of the elderly were not as strong as younger adults in tonal language speakers.

Results from previous FFR studies in older adults showed that aging may lead to the degradation of the FFR. However, poor FFR stimulus representation in the elderly might stem from age-related neural degeneration, or simply because of the overlapping response caused by the inter-stimulus interval (ISI) used in those studies. As the cumulative neural fatigue, adaption time and incomplete recovery involving hair-cell-cochlear nerve junctions and synaptic transmission in the elderly may be more inferior than that of the young, longer ISIs may be required.

The purpose of this study is to expand on our previous work on the speech-evoked FFR in older adults who speak a tonal language, and to examine the effect of different ISIs on the older adults’ FFR. Results from this study should help answer the important question of whether ISI has a different impact on older adults’ FFR compared to its impact on younger adults. It can also help bridge the gap between the laboratory research and clinical utility of FFR by recommending an appropriate length of ISI for future FFR usage in the clinical application, especially for the older population.

## Materials and Methods

This research has received approval from the Ethics Committee of Beijing Tongren Hospital Affiliated to Capital Medical University and Beijing Institute of Otolaryngology. All subjects participated in this research on their own accord. They had signed the informed consent and were compensated for their participation. All operations throughout the research did no harm on the subjects.

### Participants

**Figure 1:**
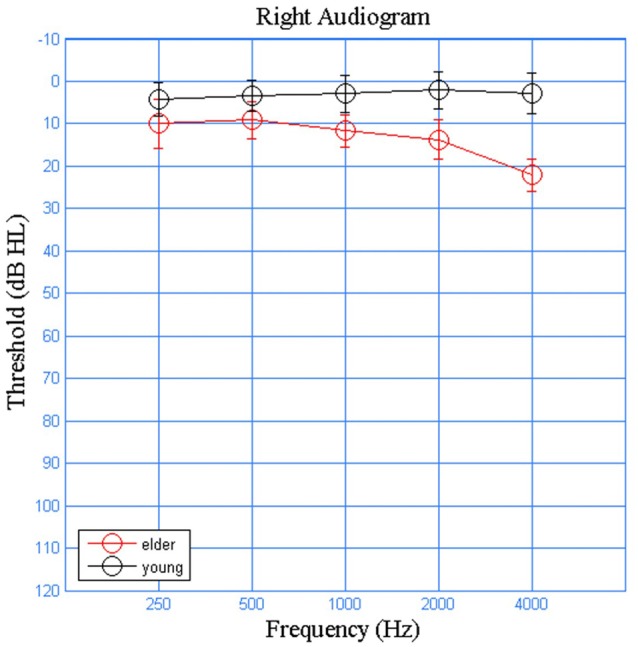
Averaged audiograms for the young (black) and older (red) participants on the right ears. Air conduction audiometric hearing threshold were denoted by circles, where error bars represent the standard deviations.

Eleven young adults (five males) and eleven elderly (four male) participants were recruited from Beijing Tongren Hospital. The younger adults were 20–26 years old (mean ± SD = 22.90 ± 2.79) and the elderly adults were 60–65 years old (mean ± SD = 62.83 ± 2.82). All participants were native speakers of Mandarin Chinese, reported no neurological or otological symptoms or illnesses. They all presented normal tympanometric measurements (Type A tympanograms and present acoustic reflexes). The older adult group had slightly elevated thresholds at higher audiometric frequencies. Still, all participants were considered to have clinically normal hearing, defined as thresholds ≤25 dB HL at octave frequencies from 250 Hz to 4000 Hz (Figure 1). All participants had normal click ABR latencies and thresholds, measured with a 100 μs click stimulus at a rate of 20.1 Hz.

### Experiment Equipment, Stimulation and Recording

All experiments were conducted in a sound-treated chamber with anechoic walls with an ambient noise ≤20 dB(A). The Smart EP instrument (Intelligent Hearing Systems, Miami, FL, USA) was used to record FFR. An electromagnetically shielded insert earphone (ER-3) was used for monoaural stimulation to the right ear. A recorded natural speech stimulus /yi/ with a falling fundamental frequency contour whose f0 ranged from 185 Hz to 135 Hz was presented at the level of 70 dB SPL. The stimulus had a duration of 250 ms, including a 5 ms rise time at the onset and a 5 ms fall time at the end. Four different ISIs (40, 80, 120 and 160 ms) were used in this study. For each ISI condition, one of the four ISIs was inserted between presentations of the speech stimulus, which resulted in an effective stimulation rate of 3.45, 3.03, 2.70 and 2.44 samples/s, respectively.

Throughout recording sessions, participants laid in bed with their eyes closed and in a calm and steady state. Three gold-plated recording electrodes were placed accordingly: high forehead as non-inverting, right mastoid as inverting and low forehead as ground (all impedances kept ≤3kΩ). The four ISI conditions were presented in random order for each participant. In each ISI condition, there were two repetitions of 2,000 accepted sweeps of the speech stimulus. The artifact rejection criteria were set at ±30 μV and the band-pass filter was set between 70 Hz and 1,500 Hz.

At the end of each experiment session, a control condition (earphone tube occluded and removed from the participant’s ear while stimulation was still present) was conducted. The recordings from the control conditions were analyzed to detect and eliminate any potential stimulus artifact.

### Data Analysis

All data were analyzed using MatLab (Mathworks, Natick, MA, USA). EEGs were filtered through a 100–1500 Hz bandpass filter with a linear phase of 500 poles. After averaging the EEG, a cross-correlation between the stimulus and recorded waveforms was carried out to identify the time shift point, that corresponds to the maximum cross-correlation value. Starting from this time point, a segment of 250 ms was extracted from the averaged data as the FFR response.

Two indices were used in this study, namely, stimulus-to-response correlation coefficient and pitch strength. The stimulus-to-response correlation coefficient, ranging from 0 to 1, is the result of cross-correlation function of stimulus waveform and FFR waveform, representing the faithfulness of pitch tracking. For discrete signals, the cross-correlation function is defined as R(n) = (1/N)*∑([x(m)*y(m+n)]), where n is time, *N* is the number of sampling points and m ranges from 0 to *N* − 1. The maximum of R(n) is defined as the stimulus-to-response correlation coefficient. The other index is the pitch strength of FFR, ranging from 0 to 1 too. It is the result of the autocorrelation function to the FFR waveform itself, representing the robustness of neural phase-locking. Pitch strength is defined as the distance between of the maximum of and the minimum of the autocorrelation function. For detailed discussion on these two indices, please refer to our previous work in Jeng and Warrington (2011) and Wang et al. (2016).

Statistical analysis was carried out with a two-way analysis of variance (ANOVA), where aging (young and older adults) and the duration of inter-stimuli intervals on FFR (40, 80, 120 and 160 ms) were evaluated as independent variables, and stimulus-to-response cross correlation coefficient and pitch strength as dependent variables.

## Results

In the older adult group, grand averaged results of stimulus-to-response correlation coefficient under the four different ISIs (40, 80, 120, 160 ms) were 0.7 ± 0.24, 0.68 ± 0.20, 0.65 ± 0.23, 0.69 ± 0.23, respectively. Similarly, grand averaged results of pitch strength under the four different ISIs were 0.38 ± 0.09, 0.45 ± 0.07, 0.39 ± 0.09, 0.39 ± 0.09, respectively. In the younger group, grand averaged results of stimulus-to-response correlation coefficient under the four different ISIs were 0.95 ± 0.03, 0.94 ± 0.04, 0.95 ± 0.07, 0.96 ± 0.03, respectively. In the same age group, grand averaged results of pitch strength under the four ISIs were 0.57 ± 0.13, 0.61 ± 0.11, 0.59 ± 0.13, 0.60 ± 0.14, respectively.

**Figure 2:**
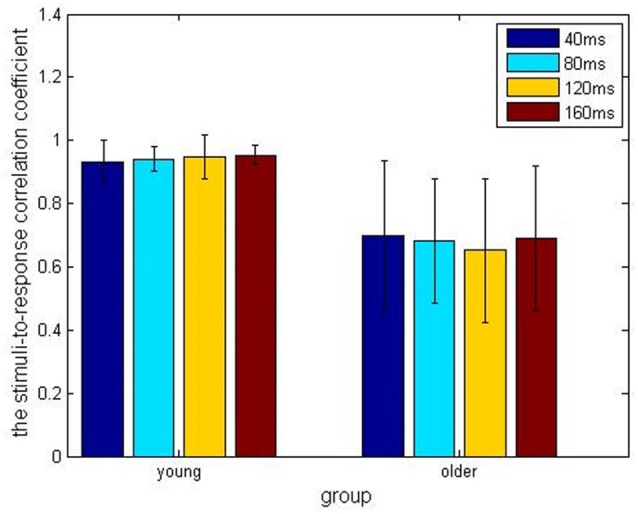
The stimulus-to-response correlation coefficients obtained under the four different inter-stimulus intervals (ISIs) of 40 ms (blue), 80 ms (cyan), 120 ms (amber) and 160 ms (crimson), respectively, and divided into the young (left) and older (right) adult groups.

**Figure 3:**
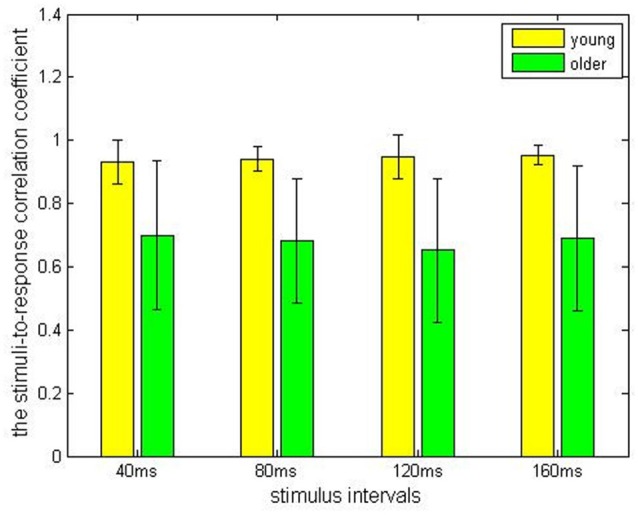
The stimulus-to-response correlation coefficients obtained from the young (yellow) and the elderly (green) groups, grouped by different ISIs.

**Figure 4:**
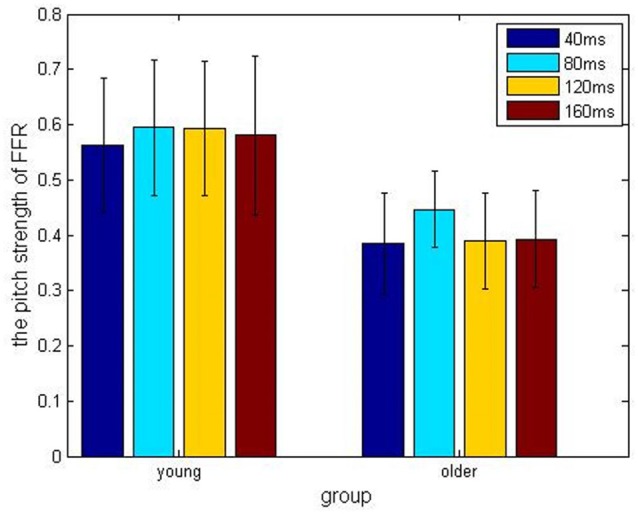
Pitch strength results obtained under different ISIs of 40 ms (blue), 80 ms (cyan), 120 ms (amber) and 160 ms (crimson), respectively, and divided into the young (left) and older (right) adult groups.

**Figure 5:**
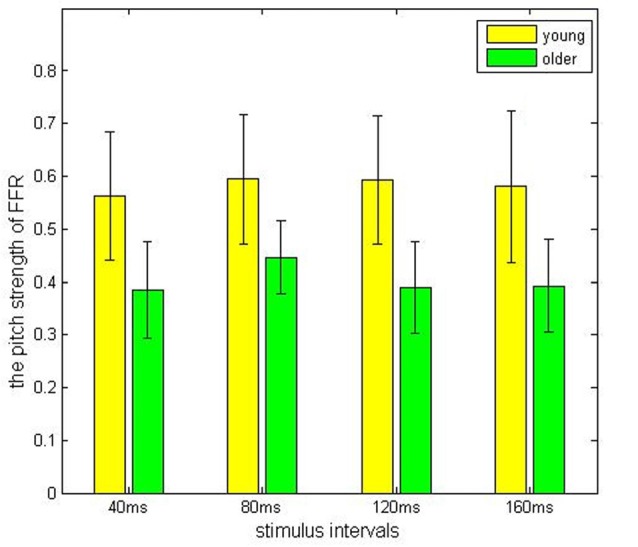
The pitch strength result obtained from the young (yellow) and the elderly (green) groups, under different ISIs.

Comparisons among the ISI levels and age groups, as well as their interactions, were calculated using two-way ANOVA. For the index stimulus-to-response correlation coefficient, two-way ANOVA showed that the four levels of ISI were not significantly different (*F*_(3,80)_ = 0.13, *p* > 0.05) from each other (Figure 2), while there was a significant different tween the two age groups (Figure 3, *F*_(1, 80)_ = 61.59, *p* < 0.01), and there was no significant interaction between factors age and ISI (*F*_(3, 80)_ = 0.08, *p* > 0.05). Similarly, for pitch strength, there was no significant difference among the four ISI levels (Figure 4, *F*_(3, 80)_ = 0.95, *p* > 0.05), while a significant difference was found between the two age groups (Figure 5, *F*_(1, 80)_ = 68.8, *p* < 0.05), and there was no significant interaction between factors age and ISI (*F*_(3, 80)_ = 0.15, *p* > 0.05).

## Discussion

### Effect of ISI in Older Adults

Results from this study suggest that different ISIs, at least for those used in the study, did not seem to impact the speech-evoked FFR. Our data revealed that although the responses were weaker than the younger adults, older adults’ FFR did not appear to be more susceptible to differing ISI lengths, some as short as 40 ms. The 40 ms ISI has been widely used in the FFR literature for Mandarin Chinese speech stimuli in younger adults (Krishnan et al., 2004, 2005; Jeng et al., 2011; Wang et al., 2016). In the current study, it did not seem to be too short for the older adults’ FFR, reflected by the two indices, stimulus-to-response correlation coefficient and the pitch strength.

This is an interesting finding in that one might think due the aging effect, the auditory pathways in older adults might need a longer ISI than their younger counterparts. Since FFR are evoked by longer and more spectro-temporally complex stimuli, which are strongly influenced by rostral brainstem and midbrain generators (Swaminathan et al., 2008), a different ISI may have been needed for older adults, as they may have decreased neural timing during transmission and processing auditory information. However, our data does not seem to support that thought. One possibility might have been that 40 ms is still long enough for the older adults to have adequate neural adaptation during the FFR recordings. Perhaps, with an ISI that is even shorter than 40 ms, one might be able to observe degraded FFR in the older groups, which can only be answered in future studies.

The result from this study is also somewhat in contrast with previous works on cortical response (Tremblay et al., 2004), where older adults showed prolonged N1 and P2 latencies when speech stimuli were presented at a higher rate. One possible explanation may be that cortical responses such as N1/P2 are compound far-field potentials that may origin from multiple neuro generators (Lightfoot, 2016). Increased stimulus rate may negatively impact the activation and transmission in some or all of those neural generators, especially in an aged auditory system. The collective result of such negative impact may manifest as a reduced evoked potential, like the N1/P2 in older adults. On the other hand, brainstem responses like the FFR have comparably simpler sources of neural activities, and that may be part of the reason why the older adults in our study did not appear to be more susceptible to the change in ISI. Our results are also a little different from Clinard and Cotter (2015), where they found that using tonal-sweep to elicit FFR, older adults seemed to have weaker responses when the stimulus rate is higher. This may be explained by the fact that the older adults in our study were all native speakers of Mandarin Chinese, and this population has been shown to have enhanced pitch processing ability and stronger FFR, which may in turn help them become less sensitive to a fast stimulus rate or a shorter ISI. Regardless, more research is needed to find out the effect on ISI or stimulus rate for different electrophysiological tests in the older adult populations, with different language backgrounds.

In short, the fact that with longer (80 ms, 120 ms and 160 ms) ISI, older adults still had poorer speech-evoked FFR compared to the younger adults, suggest that the effect of ISI, at least as short as 40 ms for speech-evoked FFR, may have played little, if any, role in the reduced response reported in this study and previous studies. Although the current study did not find significant differences in the ISIs in older adults, it does not mean that isn’t one that is too short for them. Further, there may also be one ISI where the older adults would start to have degraded response, while the younger adults still present relatively robust FFR. Future studies are need to answer these questions, as well as to compare the ISI used in FFR to those used in other auditory evoked potential tests.

### Effect of Aging on the FFR

The significant difference in older adults’ and younger adults’ FFR reaffirmed our previous work (Wang et al., 2016) and those of others (Anderson et al., 2012). These studies have demonstrated the effect of aging on the auditory system, even with “normal” audiogram, and many theories have been proposed to explain such effects. For example, elderly population have shown to have degraded temporal processing ability (Gordon-Salant and Fitzgibbons, 1999), which had been proposed to contribute to why they often complain of hearing the sounds without desired clarity, especially in the presence of background noise (Anderson et al., 2012). One explanation for poor speech discrimination and impaired speech understanding is that aging adversely affects synchronized nerve fiber firing, temporal processing and phase-locking ability (Frisina and Frisina, 1997; Burkard and Sims, 2001; Clinard et al., 2010). Other studies (Schatteman et al., 2008; Bidelman et al., 2014) showed that both brainstem and cortical speech-evoked brain responses were impacted by the aging process too, resulting in the atypical neural information processing and speech information transformation between functional levels of the auditory neural system.

Our results revealed that after controlling for the ISI, older adults still showed significantly weaker pitch processing ability at the brainstem level compared to the younger adults. Future research on FFR in older adults is needed to further investigate the underlining reason of such deficit.

### Choosing an Appropriate Inter-Stimulus-Interval for Recording Speech-Evoked FFR in Older Adults

As previously mentioned, the choice of ISI for voice-evoked FFR is an important consideration to ensure the separation of adjacent responses and completing a testing within a reasonable time frame. For recorded speech tokens such as the Mandarin Chinese syllable /yi/, which is typically 250 ms, a recording session with 2,000 repetitions can last as short as nearly 10 min with an ISI of 40 ms, and as long as 14 min with an ISI of 160 ms. In a real clinical setting, 14 min per recording session for an electrophysiological test would be too long. Fortunately, as the results of this study suggested, a 40 ms ISI yield similar responses to those longer ISIs, which means that within 10 min, a speech FFR can be obtained from typical clinical population such as older adults.

The only other study to date on the effect of ISI on speech-evoked FFR was conducted by Jeng et al. (2011), where they suggested a 35–45 ms ISI to be used in younger adults when FFR is evoked using Mandarin Chinese syllable /yi/. Our data suggest a similar recommendation: that an ISI around 40 ms is appropriate for the same FFR technique, and it can be extended to be used in both younger and older adults. With future studies, this finding fills the gap in current understanding of speech-evoked FFR, and may help paving the way for speech-evoked FFR into wilder clinical utility.

## Limitations

Although both groups had clinically normal hearing thresholds (defined as ≤25 dB HL at octave frequencies from 250 Hz to 4000 Hz), there was a difference at higher frequencies, which may have affected our result. However, had the older adults in our study actually had “better” thresholds, it would only have helped them process speech sounds better and become even less sensitive to the shorter ISIs. Regardless, since such limitations have also been reported in similar studies where auditory brainstem and cortical responses were utilized to examine the effect of aging (Tremblay et al., 2004; Anderson et al., 2012; Clinard and Cotter, 2015; Wang et al., 2016), the difference in peripheral hearing should definitely be carefully examined, considered and/or controlled for future studies in the field.

Last but least, the recommended 40 ms ISI should only be considered for speech tokens, e.g., Mandarin Chinese syllables, that are around 250 ms long. Considering that there are many types of stimuli that can licit FFR, such as CVs, tonal sweeps or even simple tones, this recommendation has its limitation in its ability to be generalized into other FFR tokens.

## Conclusion

As previously demonstrated, under different ISI conditions, older adults have weaker neurophysiological responses compared to their younger counterparts. Thus, an ISI around 40 ms could serve as a reasonable choice for future studies in the field of speech-evoked FFR in the elderly population.

## Author Contributions

DL was in charge of collecting data, analyzing statistics and writing the article. JH was in charge of designing the research and correcting the article. RD and JC were in charge of contacting the subjects and collecting data. GM were in charge of proofreading the manuscript for several times. The corresponding author SW was in charge of designing the research and correcting the article.

## Conflict of Interest Statement

The authors declare that the research was conducted in the absence of any commercial or financial relationships that could be construed as a potential conflict of interest.
